# Supplementary material for: Phylogenetic Analysis of Varicella–Zoster Virus in Cerebrospinal Fluid from Individuals with Acute Central Nervous System Infection: An Exploratory Study
Source: Viruses. 2025 Feb 19;17(2):286. doi: 10.3390/v17020286 (PMC11860453; doi:10.3390/v17020286)
Supplement: Supplementary file 1 [file viruses-17-00286-s001.zip › Fig S1.pdf]

FigS1 – Electrophoresis migration pattern in a 1.5% agarose gel. The gel shows the fragments of VZV DNA from a sample (VZV016) with amplification for ORFs 22, 38, 54 and 62 with fragment lengths of 447, 350, 222 and 419 bp, respectively. Positive controls were run in a parallel gel.

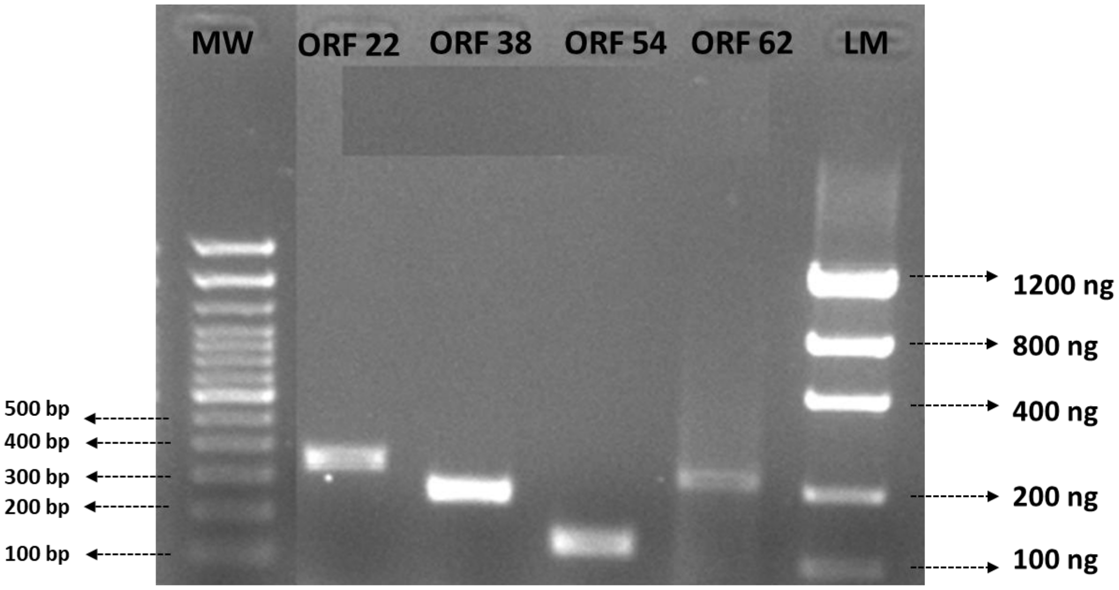

Legend: Fragments refer to conventional PCR. At right, 100 bp molecular weight (MW) and at left low mass DNA ladder for sample semi-quantification (ng).
