# Supplementary material for: Phylogenetic Analysis of Varicella–Zoster Virus in Cerebrospinal Fluid from Individuals with Acute Central Nervous System Infection: An Exploratory Study
Source: Viruses. 2025 Feb 19;17(2):286. doi: 10.3390/v17020286 (PMC11860453; doi:10.3390/v17020286)
Supplement: Supplementary file 1 [file viruses-17-00286-s001.zip › Table S1.pdf]

Table S1 – Summary of mean genetic distances between VZV isolates from Brazil and other geographic regions, clustered by clades and ORFs.

		ORF 22					
Country	Total sequences by ORF	Clade 1 or 3		Clade 2		Clade 5	
	n	n	MGD	n	MGD	n	MGD
China	71	0	1,24E-02	71	1,4E-03	0	-
Germany	21	14	7,45E-04	2	1,7E-03	5	0,00E+00
India	41	2	3,66E-03	3	0,0E+00	36	9,39E-05
Iran	19	2	2,60E-04	1	0,0E+00	16	0,00E+00
Ireland	0	0	-	0	-	0	-
Laos	22	0	8,65E-03	20	0,0E+00	2	0,00E+00
Mexico	0	0	-	0	-	0	-
Pakistan	1	0	3,67E-03	0	-	1	0,00E+00
Russia	1	1	2,60E-04	0	-	0	-
South Korea	70	1	2,60E-04	69	3,9E-04	0	-
Spain	2	2	2,60E-04	0	-	0	-
USA	63	4	1,11E-03	28	2,4E-04	31	4,37E-04
**Brazil**	19(6*)	13(2*)	-	2(2*)	-	4(*2)	-
Total (n)	330	39		196		95	

*Brazilian VZV sequences generated in this study

	ORF 38								
	Total sequences by ORF	Clade 1 or 3		Clade 2		Clade 5		Clade 6**	
	n	n	MGD	n	MGD	n	MGD	n	MGD
China	4	0	-	4	-	0	-	0	-
Germany	20	14	2,72E-03	2	6,44E-03	4	0E+00	0	-
India	9	1	2,46E-03	0	3,66E-03	8	0E+00	0	-
Iran	0	0	-	0	-	0	-	0	-
Ireland	0	0	-	0	-	0	-	0	-
Laos	0	0	-	0	-	0	-	0	-
Mexico	1	0	-	0	-	0	-	1	0,00E+00
Pakistan	1	0	-	0	-	1	0E+00	0	-
Russia	1	1	2,46E-03	0	-	0	-	0	-
South Korea	24	1	2,46E-03	23	4,42E-03	0	-	0	-
Spain	2	2	2,46E-03	0	-	0	-	0	-
USA	50	4	2,46E-03	14	4,36E-03	31	5E-04	1	0,00E+00
**Brazil**	23(10*)	16(5*)	-	1(1*)	-	5(*3)	-	1(*1)	-
Total (n)	135	39		44		49		3	

*Brazilian VZV sequences generated in this study

**There were not previous Brazilian ORF62 sequences published

	Total sequences by ORF	ORF 54							
		Clade 1 or 3		Clade 2		Clade 5		Clade 6**	
	n	n	MGD	n	MGD	n	MGD	n	MGD
China	4	0	-	4	0,00E+00	0	-	0	-
Germany	19	13	2,11E-03	2	0,00E+00	4	4,72E-03	0	-
India	8	1	2,79E-04	0	-	7	4,72E-03	0	-
Iran	0	0	-	0	-	0	-	0	-
Ireland	2	2	5,04E-03	0	7,16E-03	0	-	0	-
Laos	0	0	-	0	-	0	-	0	-
Mexico	1	0	-	0	-	0	-	1	0,00E+00
Pakistan	1	0	-	0	-	1	4,72E-03	0	-
Russia	1	1	2,79E-04	0	-	0	-	0	-
South Korea	24	1	2,79E-04	23	0,00E+00	0	-	0	-
Spain	2	2	2,79E-04	0	-	0	-	0	-
USA	64	4	2,79E-04	28	5,10E-04	31	1,57E-03	1	0,00E+00
**Brazil**	25(12*)	17(5*)	-	2(2*)	-	5(*3)	-	1(*1)	-
Total (n)	151	41		59		48		3	

*Brazilian VZV sequences generated in this study
**There were not previous Brazilian ORF62 sequences published

	Total sequences by ORF	ORF 62					
		Clade 1 or 3		Clade 2		Clade 5	
	n	n	MGD	n	MGD	n	MGD
China	4	0	-	4	1,3E-03	0	-
Germany	18	11	1,34E-03	3	3,6E-03	4	0,0E+00
India	9	1	1,41E-03	0	-	8	0,0E+00
Iran	0	0	-	0	-	0	-
Ireland	0	0	-	0	-	0	-
Laos	12	0	-	12	7,3E-04	0	-
Mexico	0	0	-	0	-	0	-
Pakistan	1	0	-	0	-	1	6,1E-03
Russia	1	1	1,41E-03	0	-	0	-
South Korea	24	1	5,25E-03	23	6,3E-03	0	-
Spain	2	2	3,33E-03	0	-	0	-
USA	63	4	2,33E-03	28	6,7E-04	31	3,8E-04
**Brazil**	6(6*)	2(2*)	-	2(2*)	-	2(2*)	-
Total (n)	140	22		72		46	

*Brazilian VZV sequences generated in this study
