# Supplementary material for: Phylogenetic Analysis of Varicella–Zoster Virus in Cerebrospinal Fluid from Individuals with Acute Central Nervous System Infection: An Exploratory Study
Source: Viruses. 2025 Feb 19;17(2):286. doi: 10.3390/v17020286 (PMC11860453; doi:10.3390/v17020286)
Supplement: Supplementary file 1 [file viruses-17-00286-s001.zip › Table S2.pdf]

Table S2. Test of the Homogeneity of Substitution Patterns Between Sequences.

A Disparity Index test to assess whether sequences have evolved with the same pattern of substitution (Kumar and Gadagkar. Genetics, 2001,158(3)). A Monte Carlo test (500 replicates) was used to estimate the P-values, which are shown above the diagonal. P-values smaller than 0.05 are considered significant (marked with yellow highlights). Evolutionary analyses were conducted in MEGA11. 1- Comparasion among Brazilian sequences. 2- Comparision among Brazilian and non-Brazilian sequences.

1	VZV 001	VZV 004	VZV 012	VZV 015	VZV0 16	VZV 018	VZV 020	VZV 021	VZV 023	VZV 026	VZV0 27	VZV 030	Marituba.BRA/30.12/1or3	Belem.BRA/26.12/1or3	Belem.BRA/18.12/1or3	Ananindeua.BRA/18.12/1or3	Ananindeua.BRA/18.12(2)/1or3	Belem.BRA/51.11/1or3	Ananindeua.BRA/18.12(3)/1or3	Ananindeua.BRA/26.12/5	Ananindeua.BRA/10.12/1or3	Ananindeua.BRA/18.12/5	Belem.BRA/08.12/1or3	Ananindeua.BRA/28.12/1or3	Ananindeua.BRA/07.12/1or3
VZV001																									
VZV004	0,32																								
VZV012	1,00	0,24																							
VZV015	1,00	1,00	1,00																						
VZV016	0,08	1,00	0,11	0,39																					
VZV018	0,25	1,00	1,00	1,00	1,00																				
VZV020	1,00	1,00	1,00	1,00	1,00	1,00																			
VZV021	0,23	0,07	1,00	1,00	0,26	1,00	1,00																		
VZV023	1,00	0,22	1,00	1,00	0,05	0,24	1,00	0,08																	
VZV026	1,00	1,00	1,00	0,06	0,08	0,05	1,00	0,01	1,00																
VZV027	1,00	0,24	1,00	1,00	0,08	1,00	1,00	1,00	1,00	1,00															
30_VZV	1,00	1,00	1,00	1,00	1,00	1,00	1,00	1,00	1,00	0,25	1,00														
Marituba.BRA/30.12/1or3	0,01	0,22	1,00	1,00	0,18	1,00	1,00	1,00	0,05	0,00	1,00	1,00													
Belem.BRA/26.12/1or3	0,01	0,21	1,00	1,00	0,18	1,00	1,00	1,00	0,10	0,00	1,00	1,00	1,00												
Belem.BRA/18.12/1or3	0,01	0,23	1,00	1,00	0,14	1,00	1,00	1,00	0,08	0,01	1,00	1,00	1,00	1,00											
Ananindeua.BRA/18.12/1or3	0,01	0,25	1,00	1,00	0,17	1,00	1,00	1,00	0,09	0,01	1,00	1,00	1,00	1,00	1,00										
Ananindeua.BRA/18.12(2)/1or3	0,00	0,21	1,00	1,00	0,16	1,00	1,00	1,00	0,08	0,01	1,00	1,00	1,00	1,00	1,00	1,00									
Belem.BRA/51.11/1or3	0,08	0,25	1,00	1,00	0,08	1,00	1,00	1,00	1,00	0,10	1,00	1,00	1,00	1,00	1,00	1,00	1,00								
Ananindeua.BRA/18.12(3)/1or3	0,01	0,19	1,00	1,00	0,16	1,00	1,00	1,00	0,07	0,01	1,00	1,00	1,00	1,00	1,00	1,00	1,00	1,00							
Ananindeua.BRA/26.12/5	1,00	0,23	1,00	1,00	0,09	1,00	1,00	1,00	1,00	1,00	1,00	1,00	1,00	1,00	1,00	1,00	1,00	1,00	1,00						
Ananindeua.BRA/10.12/1or3	0,08	0,25	1,00	1,00	0,09	1,00	1,00	1,00	1,00	0,08	1,00	1,00	1,00	1,00	1,00	1,00	1,00	1,00	1,00	1,00					
Ananindeua.BRA/18.12/5	1,00	0,24	1,00	1,00	0,08	1,00	1,00	1,00	1,00	1,00	1,00	1,00	1,00	1,00	1,00	1,00	1,00	1,00	1,00	1,00	1,00				
Belem.BRA/08.12/1or3	0,10	0,24	1,00	1,00	0,04	1,00	1,00	1,00	1,00	0,09	1,00	1,00	1,00	1,00	1,00	1,00	1,00	1,00	1,00	1,00	1,00	1,00			
Ananindeua.BRA/28.12/1or3	0,00	0,22	1,00	1,00	0,16	1,00	1,00	1,00	0,08	0,01	1,00	1,00	1,00	1,00	1,00	1,00	1,00	1,00	1,00	1,00	1,00	1,00	1,00		
Ananindeua.BRA/07.12/1or3	0,07	0,21	1,00	1,00	0,09	1,00	1,00	1,00	1,00	0,08	1,00	1,00	1,00	1,00	1,00	1,00	1,00	1,00	1,00	1,00	1,00	1,00	1,00	1,00	

**2**	VZV001	VZV012	VZV015	VZV020	VZV018	VZV023	VZV026	VZV027	VZV004	VZV021	VZV030	VZV016	JN704710.1	OQ723678.1	OQ427944.1	MH709321.1	MH709362.1	OQ835716.1	OQ454913.1	JQ972913.1	JF306641.2	MT370829.1	MF004348.1	MH709339.1
VZV001																								
VZV012	1,00																							
VZV015	1,00	1,00																						
VZV020	1,00	1,00	1,00																					
VZV018	1,00	1,00	1,00	1,00																				
VZV023	1,00	1,00	1,00	1,00	0,24																			
VZV026	1,00	1,00	0,10	1,00	0,05	1,00																		
VZV027	1,00	1,00	1,00	1,00	1,00	1,00	1,00																	
VZV004	1,00	0,25	1,00	1,00	1,00	1,00	1,00	0,25																
VZV021	1,00	1,00	1,00	1,00	1,00	0,07	0,01	1,00	0,07															
VZV030	1,00	1,00	1,00	1,00	1,00	1,00	0,25	1,00	1,00	0,26														
VZV016	1,00	0,25	1,00	1,00	1,00	1,00	0,26	0,24	1,00	0,23	1,00													
JN704710.1	1,00	0,16	1,00	1,00	1,00	0,25	0,06	1,00	1,00	1,00	1,00	1,00												
OQ723678.1	1,00	1,00	1,00	1,00	1,00	1,00	1,00	1,00	0,30	1,00	1,00	0,21	1,00											
OQ427944.1	1,00	1,00	1,00	1,00	1,00	1,00	1,00	1,00	0,28	1,00	1,00	0,23	1,00	1,00										
MH709321.1	1,00	1,00	1,00	1,00	1,00	1,00	1,00	1,00	0,06	1,00	1,00	0,05	1,00	1,00	1,00									
MH709362.1	1,00	1,00	1,00	1,00	1,00	1,00	1,00	1,00	0,22	1,00	1,00	0,25	1,00	1,00	1,00	1,00								
OQ835716.1	0,35	0,05	1,00	1,00	1,00	0,24	0,07	1,00	1,00	1,00	1,00	1,00	1,00	1,00	1,00	0,25	1,00							
OQ454913.1	0,32	0,05	1,00	1,00	1,00	0,24	0,06	1,00	1,00	1,00	1,00	1,00	1,00	1,00	1,00	0,28	1,00	1,00						
JQ972913.1	1,00	1,00	1,00	1,00	1,00	0,09	0,00	1,00	0,38	1,00	1,00	1,00	1,00	1,00	1,00	1,00	1,00	1,00	1,00					
JF306641.2	0,02	0,03	1,00	1,00	1,00	1,00	1,00	0,00	0,01	0,01	0,23	0,04	0,02	0,01	0,01	0,00	0,01	0,01	0,01	0,02				
MT370829.1	0,11	0,04	1,00	1,00	1,00	1,00	0,26	0,05	1,00	0,04	1,00	1,00	0,29	0,05	0,06	0,01	0,03	0,21	0,27	0,30	1,00			
MF004348.1	0,22	0,05	1,00	1,00	1,00	1,00	0,23	0,24	1,00	0,22	1,00	1,00	1,00	0,25	0,25	0,06	0,24	1,00	1,00	1,00	0,04	1,00		
MH709339.1	0,38	0,17	1,00	1,00	1,00	1,00	1,00	0,32	1,00	0,10	1,00	1,00	1,00	1,00	1,00	0,20	1,00	0,22	0,26	1,00	0,00	1,00	1,00	
